# A Novel Small Form-Factor Handheld Optical Coherence Tomography Probe for Oral Soft Tissue Imaging

**DOI:** 10.3390/mi15060742

**Published:** 2024-05-31

**Authors:** Alok K. Kushwaha, Minqi Ji, Sneha Sethi, Lisa Jamieson, Robert A. McLaughlin, Jiawen Li

**Affiliations:** 1 Faculty of Sciences, Engineering and Technology, The University of Adelaide, Adelaide, SA 5005, Australia; minqi.ji@student.adelaide.edu.au (M.J.); jiawen.li01@adelaide.edu.au (J.L.); 2 Institute for Photonics and Advanced Sensing, The University of Adelaide, Adelaide, SA 5005, Australia; robert.mclaughlin@adelaide.edu.au; 3 Faculty of Health and Medical Sciences, The University of Adelaide, Adelaide, SA 5005, Australia; sneha.sethi@adelaide.edu.au (S.S.); lisa.jamieson@adelaide.edu.au (L.J.)

**Keywords:** medical imaging, optical coherence tomography, probe, oral soft tissue, optical fiber

## Abstract

Tissue imaging is crucial in oral cancer diagnostics. Imaging techniques such as X-ray imaging, magnetic resonance imaging, optical coherence tomography (OCT) and computed tomography (CT) enable the visualization and analysis of tissues, aiding in the detection and diagnosis of cancers. A significant amount of research has been conducted on designing OCT probes for tissue imaging, but most probes are either heavy, bulky and require external mounting or are lightweight but straight. This study addresses these challenges, resulting in a curved lightweight, low-voltage and compact handheld imaging probe for oral soft tissue examination. To the best of our knowledge, this is the first curved handheld OCT probe with its shape optimized for oral applications. This probe features highly compact all-fiber optics with a diameter of 125 μm and utilizes innovative central deflection magnetic actuation for controlled beam scanning. To ensure vertical stability while scanning oral soft tissues, the fiber was secured through multiple narrow slits at the probe’s distal end. This apparatus was encased in a 3D-printed angular cylinder tube (15 mm outer diameter, 12 mm inner diameter and 160 mm in length, weighing < 20 g). An angle of 115° makes the probe easy to hold and suitable for scanning in space-limited locations. To validate the feasibility of this probe, we conducted assessments on a multi-layered imaging phantom and human tissues, visualizing microstructural features with high contrast.

## 1. Introduction

Oral cancer is the 16th most common cancer in the world [1]. Delayed diagnosis usually results in a poor prognosis and requires intensive invasive management strategies. The delay in diagnosis is largely due to a lack of access to health care facilities, a shortage of trained health professionals [2], poor health literacy and awareness [3], and poor imaging infrastructure [4]. Current real-time imaging techniques such as 2D X-ray imaging, X-ray computed tomography (CT) and ultrasound have limitations in visualizing oral lesions. Although recent advances have been made by utilizing machine learning to improve disease diagnosis and prognosis [5,6], CT requires undesirable exposure to radiation and has poor soft tissue differentiation, while ultrasound suffers poor resolution and sensitivity. These imaging modalities often fail to image fine details or slight alterations in tissue structure or texture [7]. There is a clinical need for alternative solutions that can provide early, non-invasive and sensitive insight into oral epithelial subsurface cellular and tissue changes [8].

Optical techniques offer non-invasive or minimally invasive methods to aid in the screening, diagnosis and monitoring of lesions. This can provide valuable information to clinicians for early detection and intervention, potentially improving outcomes for patients with cancer or pre-cancerous conditions. These approaches utilize light to examine tissues and identify abnormalities. Optical coherence tomography (OCT) [9] is one such technique that uses near-infrared light to create high-resolution cross-sectional images of tissues with a spatial resolution of 5–15 μm, helping to visualize tissue layers and identify abnormal structures [10]. OCT has been shown to aid in evaluating oral lesions such as leucoplakia and lichen planus [11,12]. A significant amount of research has been conducted on designing handheld OCT probes for tissue imaging [11,12,13,14]. However, none of these probes were a suitable shape for use in the examination of the oral cavity. The distal imaging aspect of each probe was straight and rigid, making it impractical for navigating the limited, curved spaces in the mouth and entrance to the upper airway. This leads to discomfort for the patient as well as difficulty for the clinician to examine due to the area’s inadequate accessibility. Clinical tools developed for the oral cavity are typically curved [15], which assists the healthcare professional in accessing difficult-to-see areas including the back of the throat, the floor of the mouth and retromolar areas.

In this study, we have developed an ultra-lightweight handheld OCT probe for imaging oral soft tissue. The curved probe casing has been fabricated by 3D printing an angular cylindrical tube containing the distal focusing optics and actuation mechanism for the scanning fiber. Clear resin (photopolymer) was used for 3D printing the probe casing due to its lightweight property. Due to its angular shape and lightweight features, this novel probe has a gimbaling effect that makes it easy to hold and suitable for scanning in space-limited locations. The focusing optics are a single-mode fiber spliced with no core (NC) and graded index (GRIN) fibers in order to generate a nearly collimated beam [16] that is suitable for OCT imaging. The light beam was scanned using a novel magnetic deflection mechanism. The deflection mechanism consists of a coil, a magnet and a deflection unit. We used a low-voltage and small-size amplifier and microcontroller placed outside the probe to minimize the weight of the scanning probe. To ensure vertical stability while scanning soft tissues, the fiber was passed through multiple slits positioned at various locations within the casing, providing an effective passive mechanism to reduce unwanted vertical movement. To assess the image quality of the probe, we tested this device on layers of sticky tape and human tissues. We were able to visualize microstructural features with high contrast.

## 2. Materials and Methods

**Figure 1:**
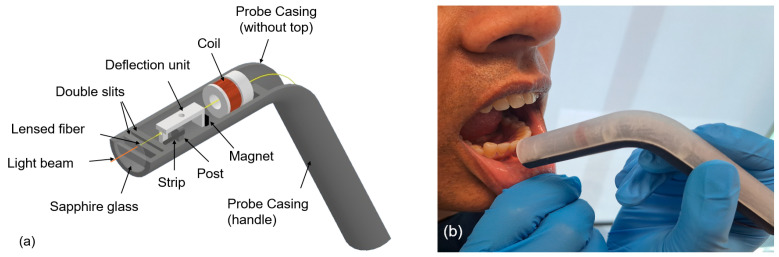
(**a**) Diagram of a handheld angular probe (isometric view) and (**b**) the in vivo application of the curved handheld probe in a human oral cavity (labial mucosa).

**Figure 2:**
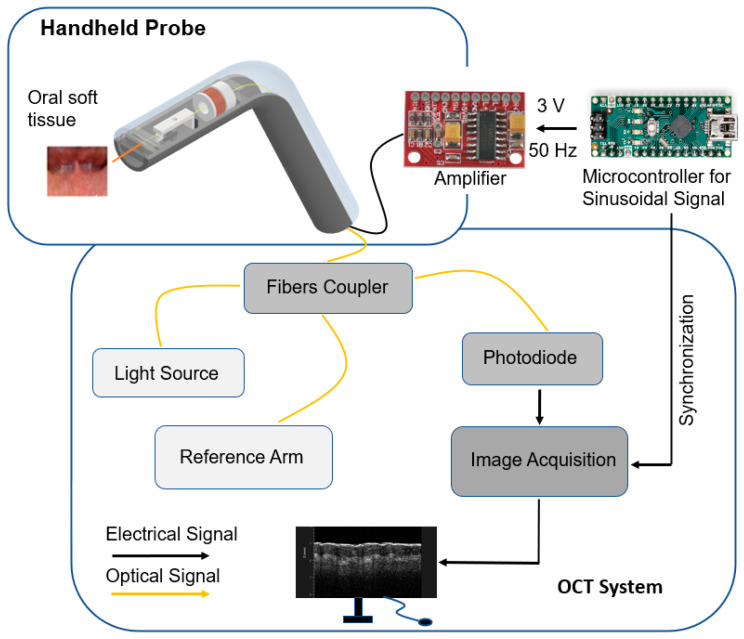
The handheld angular probe interfaced to the microcontroller and OCT system. The black color is used for electrical signals (e.g., a 3 V peak-to-peak sinusoidal signal is generated from the microcontroller to produce a magnetic field strong enough to deflect lensed fiber) and the orange color is used for optical signals (e.g., near-infrared light from the OCT system directed towards the oral soft tissue).

A diagram of a curved handheld OCT probe and its in vivo application in the human oral cavity are shown in Figure 1. The weight of the angular probe casing (including deflection mechanism) was measured to be 15.9 g. The following are the main components of the probe design: a handheld angular/curved probe casing (top and bottom parts), a deflection mechanism and a lensed fiber. The deflection mechanism consists of a copper coil, a magnet and a deflection unit. The attraction and repulsion of the magnet deflects the fiber symmetrically around the post, situated at the vertical central axis of the probe casing. As shown in Figure 2, the coil was connected to an amplifier and microcontroller to generate an alternating current signal to deflect fiber. The optical fiber was interfaced to a swept-source OCT system (central wavelength 1300 nm, Vega OCT system, ThorLabs, Newton, NJ, USA) and acquired 2D OCT B-scan images.

### 2.1. Handheld Angular Probe Casing

The handheld angular probe casing was 3D-printed (Form2, Formlabs, Somerville, MA, USA) using clear resin (photopolymer for stereolithography, SLA, Foamlabs, Somerville, MA, USA). Clear resin is a nearly transparent material with low levels of optical scattering and absorption ‘in the visible range’. The resolution selected for 3D printing was 50 μm and the size of the cylinder casing was 15 mm outer diameter, 12 mm inner diameter and 160 mm in length. 

**Figure 3:**
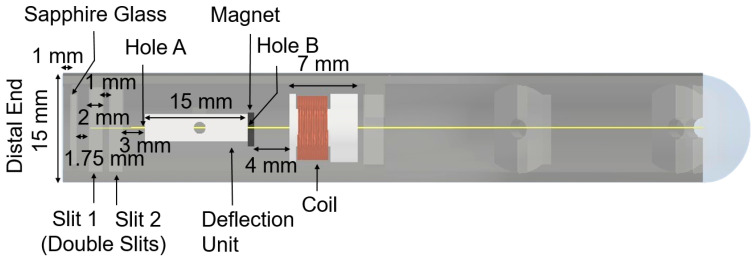
Dimensions inside the probe at the distal end. The distal end of the probe has a window slot (12 mm diameter and a groove with 1 mm depth) for the sapphire glass (8 mm diameter and 0.15 mm thickness).

A copper coil, used to generate the magnetic field, is mounted on a second 3D-printed cylinder structure (10 mm outer diameter and 4.5 mm inner diameter) and glued inside the bottom part of the probe casing. Similarly, a permanent magnet is mounted on the 3D-printed deflection unit as can be seen in Figure 3, which illustrates the relationship of these structures. 

The distances/widths that detail the location of each substructure, as shown in Figure 3, include the following:
Distance between the lensed fiber and the sapphire glass:1 mmDistance between the sapphire glass and the first slit:1.75 mmWidth of each slit:2 mmDistance between the double slits:1 mmDistance between the double slits and the deflection unit:3 mmDistance between the magnet and the coil:4 mm

These distances were empirically optimized to ensure that the fiber aligns through the center of the coil’s structure and the two holes (A and B) in the deflection unit and deflects symmetrically around the central axis of the probe. 

### 2.2. Deflection Mechanism

The deflection mechanism consists of a copper coil (60 turns × 0.25 mm diameter), a permanent magnet (neodymium block magnet—5 mm × 3 mm × 1 mm, N50) and a deflection unit (3D-printed). The number of turns of the coil was selected to be 60 so as to generate sufficient magnetic field to deflect the magnet and match the output impedance of the amplifier.

**Figure 4:**
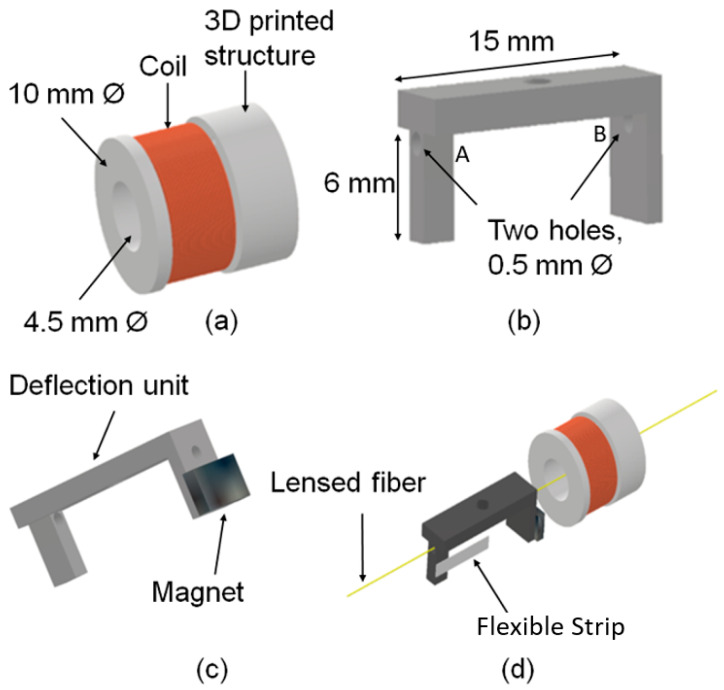
(**a**) The coil (diameter: 0.25 mm; number of turns: 60) mounted on a 3D-printed structure of 10 mm outer diameter and 4.5 mm internal diameter, (**b**) the deflection unit with two holes with a diameter of 500 μm to pass through the lensed fiber with a diameter of 125 μm, (**c**) the deflection unit with a magnet and (**d**) the assembly of the deflection unit, the magnet, the flexible strip, the lensed fiber and the coil.

The two holes (labeled A and B in Figure 4) of the deflection unit have a diameter of 500 μm. This is sufficient to hold the optical fiber (a diameter of 250 μm). 

The neodymium block magnet is attached to the deflection unit, as shown in Figure 4c such that once an alternating current signal (3 V peak-to-peak, 50 Hz) is applied to the copper coil, it generates a sufficient magnetic field to actuate the position of the optical fiber.

The attraction and repulsion of the magnet help the optical fiber to deflect symmetrically around the post, situated at the vertical central axis of the probe casing, as can be seen in Figure 1 and Figure 3. This post is attached to the deflection unit with the help of a flexible thin strip (2 mm width × 6 mm length × 0.25 mm thickness) as shown in Figure 4d. The dimension of the post is 3 mm in width, 4 mm in height and 2 mm in thickness to provide sufficient support to the strip. The purpose of the post is to act as a pivot around which deflection occurs with the help of the flexible strip. Note that the flexible strip has no direct physical contact with the fiber.

### 2.3. Lensed Fiber

The lensed fiber emits a nearly collimated beam. This is fabricated by splicing 350 μm no-core fiber and 150 μm GRIN fiber onto a single-mode fiber of length 2 m, which is similar to the design described in [11]. The lensed fiber passes through the double slits (Figure 3). The vertical gap provided between the double slits is 500 μm in order to maintain the stability of the fiber during scanning. This configuration allows the fiber to scan laterally, acquiring a 2D OCT B-scan. The fiber is placed 1 mm away from the sapphire glass, mounted at a 4° angle to the central axis of the probe. The angle was chosen to minimize back reflections from the air–window interface. 

### 2.4. Back-End System

A low-voltage (5 V) amplifier (2-channel 3W PAW8403 Audio Amplifier, Wiltronics, Victoria, Australia) and microcontroller-Arduino Nano V3.0 are used to generate a nearly sinusoidal signal of 3 V peak-to-peak. The zero crossing of a sinusoidal signal generated through the microcontroller was utilized to trigger the system. The lensed fiber was interfaced to a swept-source OCT system–Vega Series [17] with a 100 kHz sweep rate, a 1300 nm center wavelength and a 95 nm sweep range. The OCT system employs a microelectromechanical system (MEMS) tunable vertical-cavity surface-emitting laser (VCSEL) swept laser as its light source for generating long-range OCT measurements, with an imaging depth of 11 mm (in air). This long-range allows for the potential to image human tissue without contact if needed. The axial and lateral resolutions of the handheld probe with the OCT system were 15 μm and 20 μm in air, respectively, which were comparable with those reported in Reference [11]. The sensitivity of our system was 102 dB. The scanning rate of the novel handheld probe was 50 Hz. Two-dimensional cross-sectional images were obtained using a 4.5 mm field of view (FOV) with 1000 A-scans for each image (B-scan). The FOV was measured by scanning a calibrated object with periodic lines.

## 3. Results

**Figure 5:**
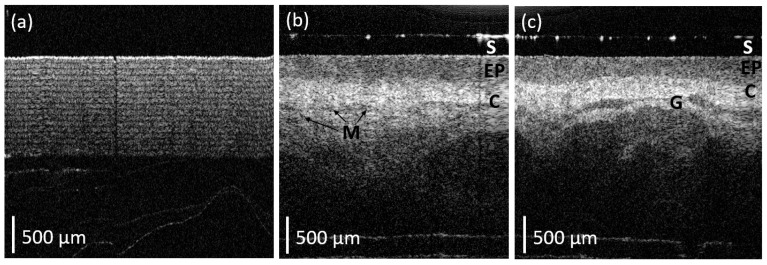
(**a**) Image of a sticky tape phantom object, which has more than 15 layers of sticky tape. (**b**,**c**) In vivo OCT cross-section of the normal human oral soft tissue. C: connective tissue; EP: epithelium; G: glandular structures; M: micro-vessels; S: sapphire glass cover.

The handheld angular probe was tested on a phantom object comprising multiple layers of sticky tape, as well as on human tissues, as illustrated in Figure 5. The resulting images clearly visualize microstructural features with high contrast. As illustrated in Figure 5a, a clear image of the sticky tape is visible for at least 1 mm into the sample. 

To demonstrate the potential clinical application of the angular handheld OCT probe, the labial mucosa of an author (a healthy volunteer of age 51 years) was imaged. The probe was disinfected by 70% ethanol before scanning oral tissue. Healthy human oral soft tissue structures (e.g., epithelium, connective tissue and micro-vessels) are also visible, as demonstrated in Figure 5b,c. In particular, the connective tissue is highly optically scattering and appears as lighter regions in the OCT B-scans. Several darker regions are also visible, allowing for the delineation of tissues with lower optical scattering such as glandular structures. The layered structure of the epithelium is clearly visible as an area of low optical backscatter at the tissue surface (the top of the image). Figure 5 illustrates the 2D B-scans images from this probe by holding it orthogonally against the labial mucosa of an oral cavity. 

The imaging resolution of a device dictates the tissue structures we can see and therefore indicates the potential applications for its clinical utility. While X-ray imaging has great utility, it lacks the spatial resolution of soft tissue differentiation required to visualize the glandular structure or tissue layer structures as seen in Figure 5 with our OCT probe.

## 4. Discussion

In this study, we have presented a novel curved, lightweight handheld OCT probe. This probe demonstrates an integration of a lensed fiber, a magnetically actuated deflection mechanism, an electrical sinusoidal signal generated by microcontroller programming, and an OCT base unit to acquire OCT images. The weight of the angular probe casing is less than 20 g. The curved design and lightweight form factor of this probe makes it novel in contrast to previous OCT probes designed for use in the oral cavity. 

The curved design utilized in this probe is highly appropriate for imaging in confined areas such as the oral cavity. This contrasts with previous probes [13,18,19,20], which were either large and required external support or which were small and straight and limited their ability to access areas deep within the oral cavity. A specific challenge in using OCT imaging for the detection of oral cancer is to gain easy access to areas deep within the oral cavity. Issues of patient compliance severely limit the use of large or long imaging probes. This is particularly significant in examining lesions near the epiglottis, deep within the oral cavity. The design presented in this paper is similar to other non-optical oral instruments commonly used in dentistry, using a curved shape to allow the distal imaging end to access deep areas that are not within the direct line-of-sight of the mouth [15]. We believe this approach provides a useful extension on previous designs and it has the potential to be extended for use in other fiber optic-based imaging modalities.

The system design presented here separates the scanhead from the microcontroller and amplifier. This architecture presents practical advantages over other lightweight OCT scanning probes [11,12], which combined the control circuitry within the probe that is inserted into the oral cavity. In particular, this simplifies the sterilization of the instrument prior to clinical use. It offers the possibility of creating a low-cost disposable scanhead that is in direct contact with the patient. This would avoid the need for re-sterilization by allowing the use of a new disposable scanhead for each patient. This reduces the clinical preparation time and makes screening for oral cancer more practical in low-resource areas that lack appropriate sterilization units. SLA-based 3D printing was utilized in this study to create the handheld angular probe casing as a proof of concept. Alternative materials or methods (such as polylactic acid through fused deposition modeling) can be used to further improve the biocompatibility and waterproof rating of the probe for future clinical applications.

## 5. Conclusions

We have presented a novel 3D-printed angular and lightweight handheld OCT probe. Our results showed that this OCT probe was able to visualize the delineation between multiple layers of sticky tape with high contrast. Similarly, in vivo scanning of human oral tissue allowed the delineation of the epithelial layer and visualization of the subsurface glandular structures and other tissues. Due to its lightweight and curved structure, it is potentially suitable for use in space-limited locations such as the oral cavity for the detection of oral cancer.

## Data Availability

The datasets generated during and/or analyzed during the current study are available from the corresponding author on reasonable request.

## Abbreviations

The following abbreviations are used in this manuscript:

OCT	Optical Coherence Tomography
GRIN	Graded Index
NC	No Core
FOV	Field of View
